# Pilot Study of Partial Tumor Ablation Using Thermal High-Intensity Focused Ultrasound (HIFU) in Feline Soft Tissue Sarcomas

**DOI:** 10.3390/ani16101530

**Published:** 2026-05-16

**Authors:** Lauren Ruger, Ester Yang, Sheryl Coutermarsh-Ott, Marlie Nightengale, Andy Hsueh, Elliana R. Vickers, Brittany Ciepluch, Eli Vlaisavljevich, Nikolaos Dervisis, Shawna Klahn

**Affiliations:** 1 Department of Biomedical Engineering, Virginia Polytechnic Institute and State University, Blacksburg, VA 24061, USA; larnold24@vt.edu (L.R.); ellianarv@vt.edu (E.R.V.); eliv@vt.edu (E.V.); 2 Department of Clinical Sciences & Advanced Medicine, University of Pennsylvania School of Veterinary Medicine, Philadelphia, PA 19104, USA; estery@upenn.edu; 3 Virginia Tech Animal Cancer Care and Research Center, Virginia-Maryland College of Veterinary Medicine, Roanoke, VA 24016, USA; mnight1@vt.edu (M.N.); andy.hsueh@thrivepet.com (A.H.); brittanyciepluch@gmail.com (B.C.); ndervisi@purdue.edu (N.D.); 4 Department of Biomedical Sciences and Pathobiology, Virginia Polytechnic Institute and State University, Blacksburg, VA 24061, USA; slc2003@vt.edu; 5 Department of Small Animal Clinical Sciences, Virginia-Maryland College of Veterinary Medicine, Blacksburg, VA 24060, USA; 6 Graduate Program in Translational Biology, Medicine and Health, Virginia Polytechnic Institute and State University, Roanoke, VA 24016, USA; 7 Department of Veterinary Clinical Sciences, Purdue University College of Veterinary Medicine, West Lafayette, IN 47907, USA

**Keywords:** high-intensity focused ultrasound, thermal ablation, immunotherapy, feline, oncology, soft tissue sarcoma

## Abstract

Soft tissue sarcomas (STS) are locally invasive and aggressive tumors that occur spontaneously in humans, dogs, and cats. High-intensity focused ultrasound (HIFU) is a non-invasive ablation technology that has been explored in canine but not feline STS. The objective of this pilot study was to determine the in vivo safety and feasibility of HIFU ablation for feline STS and to investigate the impact of HIFU on the acute immunological response. Client-owned cats diagnosed with spontaneous STS were recruited. Computed tomography (CT) scans of the chest, abdomen, and tumor were performed prior to treatment for staging and treatment planning. A commercially available HIFU unit (Echopulse, Theraclion, Malakoff, France) was used to target portions of solid tumors before standard-of-care surgical resection. Ablation efficacy and local immunological response were characterized using histopathological and immunohistochemical assessments. Acute safety was monitored with physical examinations, owner reports, and CBC/serum biochemistry. Multiplex serum cytokine levels were used to evaluate the systemic immune response. A total of three cats diagnosed with STS were recruited and treated. No significant adverse events attributed to HIFU treatment were noted in this pilot study. In treated areas, hemorrhage as well as coagulative and lytic necrosis were observed microscopically and were more extensive than in untreated tissues. There was a statistically significant difference in the level of serum MCP-1 after HIFU treatment, but no significant changes in any other analytes. No differences in the infiltration of CD3-, CD79a-, or IBA1-positive cells were noted between treated and untreated samples. Overall, findings suggested that HIFU may offer a viable alternative to conventional therapies for feline STS, with pilot results showing effective tumor ablation in cats with STS without significant adverse events. Some preliminary evidence of immunomodulation following treatment was observed, but HIFU as an immunotherapeutic treatment option needs to be further investigated.

## Simple Summary

High-intensity focused ultrasound (HIFU) is an emerging, non-invasive ablation technique in which high-pressure ultrasound waves are applied to a fixed point within the body to destroy tissue. Soft tissue sarcomas (STS) are locally invasive and aggressive tumors that occur spontaneously in humans, dogs, and cats. Pilot studies have demonstrated the safety and feasibility of HIFU for canine, but not feline, STS. In the current pilot study, three cats with STS were treated using a commercially available HIFU unit (Echopulse, Theraclion, Malakoff, France) before standard-of-care surgical resection of the tumor. All three cats tolerated treatment well without significant adverse events, and post-resection analyses confirmed effective tissue destruction was achieved in the treated tumor regions. Some evidence of immunological changes following HIFU was observed, but findings were limited by the small cohort of cats and further investigation is needed. Overall, the results of this pilot study demonstrate HIFU’s potential as a non-invasive treatment for STS in cats and lay the foundation for future work investigating HIFU as an alternative to surgery for feline STS.

## 1. Introduction

Feline soft tissue sarcomas (STS), including feline injection site sarcomas (fISS), are rapidly growing tumors with low metastatic potential but locally aggressive behavior [1]. Complete surgical excision remains the treatment of choice. Surgical margins of 4–5 cm result in 95% of tumors having complete, tumor-free histologic margins. However, due to tumor size at diagnosis and the tumor’s infiltrative nature, achievement of gross surgical margins of this size is feasible in only a small percentage of patients. In contrast, surgical margins of 2–3 cm are more clinically feasible, but result in only 50% of tumors exhibiting complete, tumor-free histologic margins [2]. Thus, multimodal local therapy is commonly recommended, such as radiation therapy prior to surgery or as an adjunct following incomplete or narrow resection [3]. Local tumor control remains the life-limiting problem for most cats, as 28% to 45% of tumors recur despite conventional multimodal local therapy [1]. Additional local therapies are needed to improve patient outcomes.

High-intensity focused ultrasound (HIFU) is an emerging, non-invasive ablation technique in which high-pressure ultrasound (US) waves are applied to a fixed point within the body to destroy tissue by either thermal or mechanical means [4]. Thermal HIFU ablation is achieved through the absorption of ultrasonic energy generated by a seconds-long, continuous HIFU pulse, which rapidly elevates the temperature in the target tissue [5]. This temperature elevation results in thermal coagulation from protein denaturation and irreversible cell damage, which is the primary mechanism for tumor cell destruction from HIFU [6]. Additionally, the US pulse is focused within a small target volume of tissue to minimize thermal injury to surrounding healthy tissues, such as skin, nerves, or vessels.

Previous studies have demonstrated that HIFU can also have an immunostimulatory effect [7,8,9]. This effect may be related to HIFU’s ability to promote immunogenic cell death. The in situ tumor debris remaining after thermal ablation can contain tumor antigens in a spectrum of denatured to non-denatured states, corresponding to the temperature reached in the focal zone [10]. These tumor antigens can then either passively enter circulation or be recognized by an antigen-presenting cell, which in turn presents them to immune effector cells. By initiating an adaptive antitumor immune response, the preserved tumor antigens may function as an in situ cancer vaccine to stimulate local and potentially systemic antitumor immune responses [11,12]. HIFU has demonstrated immunomodulation and/or immune sensitization in many preclinical models and clinical trials, including brain [13], breast [14,15], and prostate cancers [16], as well as in sarcomas [7]. Thus, thermal HIFU is a promising candidate as a less invasive and potentially immunogenic alternative to standard treatment options for feline STS.

HIFU has previously been used for the treatment of both dogs and cats with STS, showing preliminary safety and efficacy [17]. Histopathology from tumors resected post-treatment revealed well-circumscribed lesions of ablation and coagulative necrosis, and there were no procedure-related adverse events recorded. However, this HIFU ablation was guided by magnetic resonance imaging (MRI) rather than US; in both the human and veterinary medicine settings, US is far more available, accessible, and cost-effective than MRI [18]. This is even more pronounced in veterinary medicine [19,20]. Therefore, US-guided HIFU may offer cost benefits over MR-guided HIFU in the veterinary setting and warrants further exploration for comparative oncology applications.

To date, our team has treated 15 dogs with peripheral STS using US-guided HIFU, demonstrating safety, complete tumor kill in the ablation zone, infiltration of lymphocytes into the tumor microenvironment, and upregulation of genes associated with inflammation [21]. However, feline STS, and particularly fISS, represent a unique challenge for ablative therapies, including HIFU. First, the smaller body size of the cat compared to that of the dog results in more superficial tumors closer to the skin and other critical structures, posing an increased risk for off-target damage. Second, fISS behave more aggressively than other sarcomas, exhibiting extreme local invasion and recurrence [22,23,24], and may require specialized treatment strategies or protocols for complete ablation. Finally, fISS is thought to be caused by vaccination- or injection-induced inflammation, leading to a uniquely inflammatory tumor microenvironment (TME) that could potentially allow for increased immunogenicity following ablation [25].

The overarching goal of this pilot study was to explore HIFU’s potential as a novel, non-invasive treatment strategy for feline STS. In this multi-case report, three cats with STS were treated with thermal HIFU before standard-of-care surgical resection of the tumor 1 to 5 days later to allow for histopathological analysis of the ablation zone and immune characterization of the local TME. Because the safety and feasibility of HIFU in cats had not previously been established, the objective of this pilot study was to prospectively evaluate the in vivo tolerability and feasibility of HIFU treatment in cats with spontaneous STS, while the secondary objective was to characterize the acute immunological response following HIFU ablation. Ablation efficacy and local immunological response were characterized using histopathological and immunohistochemical assessments. Multiplex serum cytokine levels were used to evaluate the systemic immune response. If shown to be safe and effective, HIFU has the potential to change the landscape of oncological care for STS in companion animals, offering non-invasive and repeatable ablation without the risks and monetary costs associated with surgery. At the same time, there is significant overlap in STS subtypes, clinical behavior, and recommended therapies across all species with STS, such that cats and dogs with STS provide unique clinical-translational potential for the development of HIFU treatment in human medicine [26,27,28,29].

## 2. Materials and Methods

### 2.1. Patient Screening and Enrollment

This was a prospective, single-arm pilot study investigating HIFU treatment in cats diagnosed with STS. Client-owned cats with naturally occurring, externally accessible cutaneous or subcutaneous STS of the body wall or extremities presenting to the Virginia-Maryland College of Veterinary Medicine Animal Cancer Care and Research Center over a 15-month period (April 2021–July 2022) were evaluated for eligibility. In this study, “externally accessible” was used to describe tumors that could be visualized ultrasonographically, coupled to the HIFU treatment unit, and approached without intervening body cavities or major anatomic barriers. Owners of eligible cats were offered standard treatment options, including palliative care, and informed owner consent was obtained for all cats enrolled in the trial. The trial was approved by the College of Veterinary Medicine Hospital Board and the Virginia Tech Institutional Animal Care and Use Committee under IACUC protocol #20-180.

Feline patients with a cytologic or histologic diagnosis of malignant STS were considered for enrollment. Trial inclusion criteria included (1) a tumor diameter of ≥3 cm in the longest dimension, (2) complete tumor resectability as determined by a Diplomate and Surgical Oncology Fellow of the American College of Veterinary Surgeons (ACVS), (3) an expected patient survival of >4 weeks without treatment, and (4) owner compliance with all scheduled treatment visits. At the time of screening, patients underwent routine laboratory bloodwork plus thoracic and abdominal imaging. Cats were excluded from the trial if the tumor was deemed non-resectable, if surgical resection was declined, if the patient had definitive therapy other than surgery within the past 3 weeks, or if the cat had a co-morbidity preventing anesthesia. Conditions which would preclude anesthesia included significant cardiac, pulmonary, or renal dysfunction or alanine aminotransferase or aspartate aminotransferase values ≥ 3× the upper reference limit.

### 2.2. Evaluation Timeline

Baseline evaluation was performed within 10 days prior to HIFU treatment and included: (1) a physical examination, (2) complete blood count (CBC) and serum biochemistry tests, (3) tumor measurements using calipers and gross photographs, (4) pre-treatment biopsy, and (5) contrast-enhanced computed tomography (CT) scans (SOMATOM Confidence^®^ RT) of the thorax, abdomen, and tumor. The pre-treatment biopsy was performed outside of the planned treatment zone so as to not interfere with the treatment path. On the morning of HIFU treatment, the baseline physical exam was repeated, and tumor photographs were taken immediately prior to and immediately following treatment. One day post-treatment as well as immediately prior to surgical resection, the patient underwent another physical exam and whole blood collection, and tumor measurements and photographs were collected. Tumor resection was planned within 5 days post-treatment based on prior work with thermal HIFU in dogs with soft tissue sarcomas [21] but was open to adjustment based on the patient’s clinical needs. Surgical excision and post-operative recommendations for all feline patients were directly performed or supervised by an oncology-fellowship-trained Diplomate of the ACVS. Two weeks after surgery, patients were again examined, and the surgery site was photographed. Post-trial monitoring recommendations were at clinician discretion, including monthly physical exams and thoracic radiographs appropriate to the patient’s tumor type and stage. Patient outcome was documented by recheck examination and/or communication with the client or primary care veterinarian.

### 2.3. HIFU Treatment

Treatment was administered using a commercially available HIFU system (Echopulse, Theraclion, Malakoff, France). The system consists of a visualization and treatment unit (VTU) affixed to a robotic arm and mounted onto an electronic cabinet base. The VTU is made up of a piezoelectric treatment transducer with a coaxially aligned ultrasound imaging probe to allow for real-time monitoring of the treatment site. The treatment transducer had a single element operating at 3 MHz and a focal length of 38 mm. The imaging probe contained a linear array of 128 elements that operate at frequencies between 5 and 10 MHz.

On the day of treatment, all patients were placed under general anesthesia following standard protocols for client-owned cats; anesthesia was maintained using inhaled isoflurane throughout the procedure. Vital signs were monitored every five minutes (blood pressure, pulse, and ventilation) or fifteen minutes (oxygen saturation, carbon dioxide saturation, body temperature and cardiac arrhythmias) by a licensed veterinary technician with veterinarian oversight throughout the duration of treatment. To prepare the skin overlying the treatment area, fur was clipped and then removed using depilatory body cream (Nair Body Cream, Naircare, Ewing, NJ, USA) applied for 5–10 min. In consultation with the surgical team, an appropriate acoustic window, treatment zone, and ablation volume were identified for HIFU using baseline CT images. Pre-treatment biopsies were collected away from the HIFU target zone.

**Figure 1:**
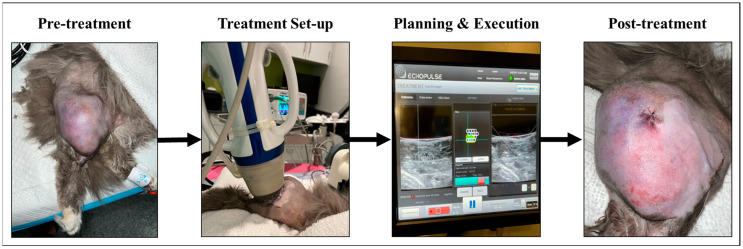
HIFU treatment day workflow for a representative patient (Patient 2). Pre-treatment, the skin overlying the tumor treatment area was prepared. During treatment set-up and planning, the VTU of the HIFU system was coupled to the patient’s tumor, and the target ablation volume was planned using the device’s custom software. Then, the treatment was executed and monitored via the device’s user interface with real-time B-mode ultrasound imaging. Treatment voxels were depicted as ellipses overlying the tumor image, with the treated locations highlighted in green in the graphic. Post-treatment, the HIFU unit was removed and patient tumors were photographed and monitored for changes. The sutures shown in the post-treatment image were from the pre-treatment biopsy away from the treatment site.

Immediately prior to treatment, a freehand US scan was performed to identify the tumor, skin, and any nearby critical structures, such as bone, nerves, and blood vessels. Then, the VTU was coupled to the patient using a balloon attached to the face of the unit and filled with degassed water and a layer of ultrasound gel (Figure 1). Water was circulated between a chiller and the balloon located on the VTU throughout treatment to reduce the risk of thermal injury to the skin.

The target ablation volume was manually delineated using the device’s treatment planning software based on real-time ultrasound imaging, pre-treatment CT images, and clinician assessment of the planned surgical field. Treatment voxels were preferentially positioned within solid portions of the tumor, while grossly cavitated or necrotic regions were avoided when possible to improve histologic interpretation of treatment-induced tissue damage.

Treatment depths ranged from 10.1 to 40.2 mm beneath the skin surface. This range reflected the technical treatment window of the HIFU system, the superficial location of feline STS, and the need to maintain an adequate distance between the focal zone and the skin surface to reduce the risk of thermal injury. Treatment depth was selected individually for each patient based on tumor size, tumor geometry, skin-to-tumor distance, and proximity to adjacent critical structures.

Each treatment voxel represented the tissue volume targeted by a single HIFU pulse and measured approximately 7.3 × 5 × 5 mm, corresponding to an estimated volume of 0.1 mL. The number and arrangement of voxels varied between patients because tumor size, shape, and safely accessible treatment volume differed among cats. Therefore, treatment plans were individualized rather than standardized to a fixed number of voxels or a fixed percentage of total tumor volume. However, the same device platform, imaging-guided planning approach, voxel definition, and safety constraints were applied across all patients to support consistency in treatment delivery. The VTU was robotically repositioned between treatment sites to treat the prescribed tumor volume, which was smaller than the total tumor volume to achieve partial ablation of the tumor. The experimental set-up and photographs of a representative tumor pre- and post-HIFU are shown in Figure 1.

### 2.4. Evaluation of Safety and Scoring of Adverse Events

Safety was monitored with physical examinations, owner reports, and CBC and serum biochemistry profiles. Adverse events (AEs) were graded according to the Veterinary Cooperative Oncology Group—Common Terminology Criteria for Adverse Events (VCOG-CTCAE v2) [30]. Severe AEs were defined as any grade 4 or 5 toxicity [30]. Thermal injuries to the skin overlying the treatment site were assessed according to the grading scheme described by Wohlsein et al. [31].

### 2.5. Evaluation of Ablation Effectiveness

Following surgical removal, the tumor was sectioned into untreated and treated regions, and gross photographs were obtained. Treatment areas were identified at gross examination through a combination of clinician communication, review of images taken at treatment, and/or inking of the skin at the time of treatment. After sectioning, all collected tissues (pre-treatment, untreated, and treated) were routinely fixed in 10% formalin for ≥24 h, embedded in paraffin, sectioned into 5 μm slices, and stained with a hematoxylin and eosin (H&E) stain to (1) confirm the diagnosis, (2) assess completeness of margins, and (3) assess the extent of HIFU-induced damage to the targeted region. Slides were reviewed to investigate microscopic features of HIFU ablation by a veterinary pathologist and Diplomate of the American College of Veterinary Pathologists (ACVP) with extensive experience evaluating ablated tumor tissue (S.C.O.).

### 2.6. Evaluation of the Local Tumor Microenvironment

Immunohistochemistry to investigate macrophage, T cell, and B cell populations was performed on formalin-fixed, paraffin-embedded pre-treatment, untreated, and treated tumor sections by a board-certified veterinary pathologist (S.C.O.). Antibodies against IBA1 (FujiFilm, Tokyo, Japan, 019-19741), CD3 (Agilent Dako, Glostrup, Denmark, A0452), and CD79a (Santa Cruz Biotechnology, Dallas, TX, USA, HM47) were used. All samples were processed on a Roche Ventana Discovery Ultra Automated Stainer using a heated Tris buffer for antigen retrieval and the Ultraview Red detection system. All slides were counterstained with hematoxylin.

### 2.7. Evaluation of the Systemic Immune Response

Whole blood and plasma samples were collected pre-HIFU and 1 day post-HIFU. Batched cytokine analysis using a commercially available Bead-Based Multiplex Assay was performed to quantify 19 separate cytokines and chemokines (FCYTMAG-20K-PMX, Millipore Sigma, Burlington, MA, USA) involved in inflammation and immune signaling. The following cytokines were measured: Fas, Flt-3L, GM-CSF, IFNγ, IL-1β, IL-2, IL-4, IL-6, IL-8, IL-12 (p40), IL-13, IL-18, KC, MCP-1, PDGF-B, RANTES, SCF, SDF-1, and TNFα. The assay was run by the University of Virginia Flow Cytometry Core (RRID: SCR_017829) according to the manufacturer’s directions. Briefly, samples were randomized on the plate, incubated overnight at 4 °C, and washed using a magnetic plate washer. Then, all standards, quality controls, and samples were analyzed in duplicate on the Luminex^®^ 200TM multiplexing instrument (Luminex Corp., Austin, TX, USA). Finally, sample analyte concentrations were determined using standard curve data from each run via Belysa^®^ curve fitting software v1.2 (Millipore Sigma, Burlington, MA, USA).

### 2.8. Statistical Analysis

The residuals of all datasets used for statistical analysis were evaluated for normal distribution with the Shapiro–Wilk test. Differences between pre- and post-HIFU cytokine levels were evaluated using paired *t*-tests for normally distributed data and Wilcoxon matched-pairs signed rank tests for residuals that did not meet a normal distribution (*p* < 0.05 on Shapiro–Wilk test). Differences between pre- and post-HIFU cytokine levels were considered statistically significant if *p* < 0.05. Descriptive statistics are reported as mean and range of values unless otherwise stated.

## 3. Results

### 3.1. Patient Population

The characteristics of the feline patients, as well as the grade, subtype, excision status, and location of the treated tumors, are summarized in Table 1 [32]. In all cats, the tumor was well visualized on the pre-treatment CT scan. Tumors generally exhibited hyperattenuating soft tissue compared to healthy muscle, with some central regions of hypoattenuation. Contrast enhancement was localized at the periphery and more infrequently at the center of the mass. Contrast-enhanced CT images of the thorax and abdomen were reviewed for evidence of distant metastatic disease, and regional lymph nodes were evaluated when included in the imaging field. No cats had metastatic disease identified on staging that precluded HIFU treatment or planned surgical resection. The histologic diagnosis of the tumors included two grade II fibrosarcomas and one grade III fibrosarcoma. After HIFU treatment, all three cats had their tumors surgically excised within 5 days as planned. Two cats had an amputation of the affected limb performed due to the location of the tumor limiting surgical margins (Patient 1 and Patient 2; 5 and 1 days post-HIFU, respectively), and one cat had wide surgical resection of the tumor (Patient 3; 1 day post-HIFU). All gross and microscopic surgical margins were free of neoplastic cells. Histologic tumor-free margins ranged from 0.4 cm to ≥2.0 cm.

All three cats were still alive at the conclusion of trial follow-up (median follow-up of 131 days, range 81–543 days). No cats were euthanized, and none were lost to follow-up. One cat diagnosed with grade III fibrosarcoma received a single dose of doxorubicin 27 days post-operatively.

### 3.2. HIFU Treatment Outcomes

All tumors were accessible to HIFU treatment. The mean treatment duration (±standard deviation) was 18.33 ± 7.5 min. An average of 22 ± 12.12 sites were treated per tumor. The mean delivered energy per tumor was 5.8 ± 3.89 kJ, with a median requested power per pulse of 35 W (range: 25–40 W) per tumor. Study deviations from the planned timing of surgical resection occurred in two cats. These deviations were related to progression of clinical signs attributable to the underlying disease and to clinical scheduling constraints, rather than to HIFU-associated toxicity. Patient 2 had pain associated with a rapidly growing tumor, was difficult to transport and medicate, and the owner had limited availability for additional visits. Patient 3 had progressive anemia before HIFU treatment, with a packed cell volume of 19% on the day of treatment compared with a hematocrit of 26% measured five days earlier by the primary veterinarian. A blood transfusion was administered during anesthesia to reduce the risk of cardiovascular decompensation during biopsy and HIFU treatment. This cat developed a mild transfusion reaction characterized by increased body temperature and was monitored overnight in the intensive care unit. Surgical resection was performed the following day. In both cats, the relevant clinical signs were present before HIFU treatment and improved after surgical removal of the tumor and supportive care. Trial protocol deviations were approved by the trial investigators and were not directly attributable to HIFU treatment.

### 3.3. Adverse Event Assessment

In this small pilot cohort, no grade 4 or 5 adverse events attributable to HIFU were observed; however, the sample size was insufficient to define the frequency or full se-verity spectrum of HIFU-associated adverse events in cats. Adverse events likely attributable to disease included lethargy, anorexia, pain, and anemia and resolved following blood transfusion and surgical resection.

In all patients, HIFU treatment was well tolerated, with no clinically significant variations from expected vital values recorded during anesthesia. During HIFU, body temperatures were maintained between 37.4 °C and 39.3 °C, and pulse rates were maintained between 90 and 185 beats per minute. Mean blood pressures ranged from 60 to 185 mmHg, with one patient (Patient 3) experiencing persistent hypotension at 40 mmHg that resolved after administering norepinephrine constant-rate infusion and increasing the blood transfusion rate. Oxygen saturation levels were maintained between 93 and 100%, and end tidal carbon dioxide levels were held at 25–44 mmHg. Other than the one instance of hypotension, which responded to treatment, no complications were reported during anesthesia or anesthetic recovery in any of the patients.

Additionally, no clinically significant AEs associated with HIFU treatment were reported through bloodwork, post-treatment physical examination, or owners’ reports. On post-treatment physical examination one day post-HIFU, the mass from one patient was reported to be warm to the touch (Patient 1), and erythema of the skin overlying the mass was recorded for all three cats. Cutaneous changes were self-limiting and did not result in a change in VCOG-CTCAE dermatologic adverse-event grade [30]. Based on the burn classification described by Wohlsein et al., the lesions were most consistent with first-degree and superficial second-degree thermal injury in two cats, and no cutaneous injury required clinical intervention prior to planned surgical resection [31].

### 3.4. Gross and Histologic Findings

**Figure 2:**
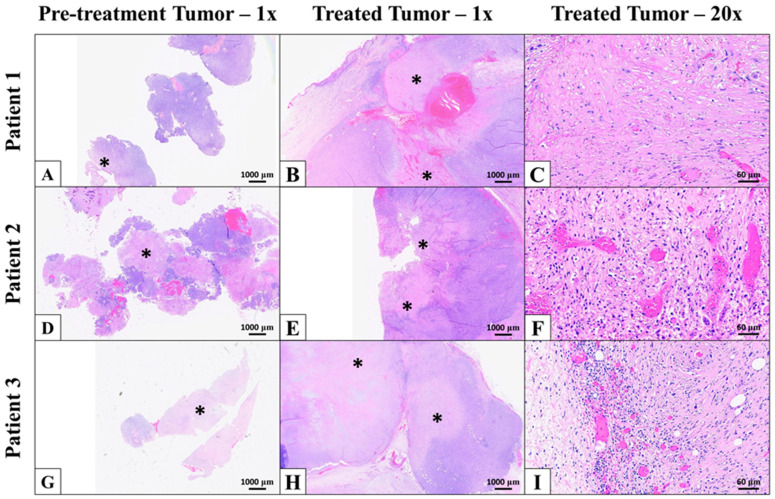
Representative histology images for patient samples collected pre- and post-HIFU. Pre-treatment samples (**A**,**D**,**G**) for all patients were characterized as densely packed spindle cell neoplasms with foci of necrosis (*) and hemorrhage. HIFU-treated samples (**B**,**C**,**E**,**F**,**H**,**I**) were similarly characterized by lytic and coagulative necrosis (*), acute hemorrhage, and fibrin deposition. For each patient, foci of necrosis were more extensive in treated samples compared to those collected pre-treatment. High-magnification images depicting areas of necrosis and hemorrhage in treated samples are shown in (**C**,**F**,**I**). Magnifications are noted in the figure headings.

Grossly, all treatment sites were characterized by foci of tissue softening suggestive of necrosis. Additionally, relatively indiscrete foci of mild hemorrhage were often appreciated. Three samples from each patient were evaluated microscopically with H&E staining: a pre-treatment sample taken from the tumor immediately prior to treatment and two samples collected after surgical resection (untreated and treated). Microscopically, all treated areas exhibited a combination of coagulative and lytic necrosis, characterized by a replacement of intact tumor cells with cellular debris, as well as varying degrees of acute hemorrhage and fibrin deposition (Figure 2B,C,E,F,H,I). In one section, necrosis of vessels was a prominent feature in the treated area. There was a moderate amount of coagulative and lytic necrosis, as well as acute hemorrhage, in pre-treatment and untreated tumors, likely secondary to tumor environment (Figure 2A,D,G). For each individual tumor, foci of necrosis and hemorrhage were more extensive in treated versus untreated samples. All treated samples had random nodules of intact tumor cells remaining in the ablation zone. The overlying skin was reported to be variably discolored and eroded to ulcerated.

### 3.5. Immunohistochemical Findings

**Figure 3:**
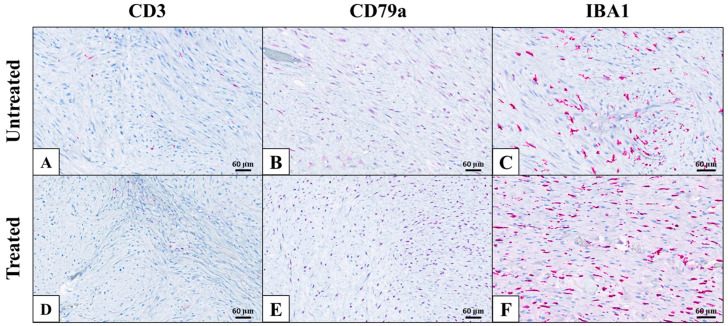
Representative immunohistochemistry images from Patient 3. Immunohistochemistry to characterize tumor microenvironment was performed on pre-treatment, untreated, and treated tumor samples. Antibodies against CD3 (**A**,**D**), CD79a (**B**,**E**), and IBA1 (**C**,**F**) were used to investigate T cell, B cell, and macrophage populations, respectively. No major changes were observed in immune cell populations from pre-treatment or untreated samples (**A**–**C**) when compared to treated samples (**D**–**F**). Generally, tumors exhibited low numbers of CD3-positive cells amongst tumor cells, rare CD79a-positive cells, and moderate numbers of IBA1-positive cells. All images were collected at 20× magnification.

To investigate immune signatures, immunohistochemistry for IBA1 (pan-macrophage marker), CD3 (pan-T cell marker) and CD79a (B cell marker) was performed on pre-treatment tumor samples as well as untreated and treated tumor samples collected after surgical resection 1 day (Patients 2 and 3) or 5 days (Patient 1) post-HIFU. Overall, all samples appeared relatively similar (Figure 3). Intact tumor cells from pre-treatment, untreated, and treated tumor samples exhibited relatively high numbers of individual and aggregated IBA1-positive cells. Low numbers of IBA1-positive cells were present in areas of necrosis in all samples exhibiting such foci regardless of treatment status. Similarly, intact tumor cells from all samples exhibited very low numbers of individual, widely scattered CD3-positive cells. One out of three samples exhibited no to extremely rare individual CD79a-positive cells in intact tumor cells. The remaining two samples exhibited nonspecific CD79a staining of tumor cells (nuclei in one sample and cytoplasm in the other), preventing adequate evaluation.

### 3.6. Serum Cytokine Analysis

**Figure 4:**
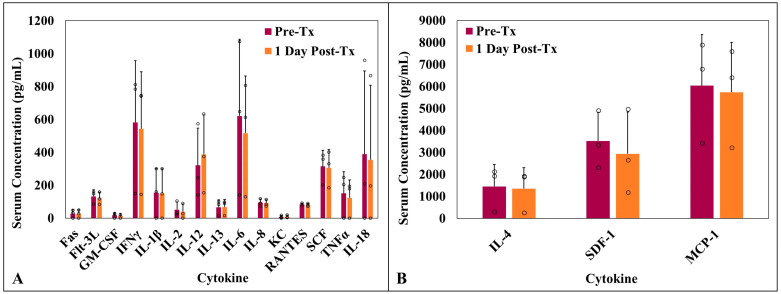
Quantified serum cytokine concentration compared pre- and 1 day post-HIFU treatment for 18 of 19 analytes. (**A**) Average concentrations for Fas, Flt-3L, GM-CSF, IFNγ, IL-1β, IL-2, IL-12, IL-13, IL-6, IL-8, KC, RANTES, SCF, TNFα, and IL-18. (**B**) Average IL-4, SDF-1, and MCP-1 serum concentrations. PDGF-B was below the minimum detection limit of the assay and excluded from the figure. A significant decrease in MCP-1 (two-tailed *p* = 0.0279) was measured after HIFU treatment; no significant changes were observed for any of the other analytes.

Serum cytokine concentrations were compared pre-treatment and one day post-HIFU for all three cats. Individual average serum cytokine concentrations were widely distributed for all analytes pre- and post-treatment. There was a statistically significant decrease in MCP-1 after HIFU treatment (two-tailed *p* = 0.0279); no significant changes were detected in the average patient serum cytokine concentrations for any of the other analytes (Figure 4). Serum cytokine concentrations for each analyte, along with the respective means and standard deviations, are reported in Table 2. Analyte PDGF-B was not detectable in any of the cats at all timepoints and was excluded from statistical analysis.

## 4. Discussion

The primary objective of this pilot study was to demonstrate preliminary safety and feasibility of HIFU treatment for feline soft tissue sarcomas (STS). In all three patients, HIFU was well tolerated, and effective ablation of targeted tumor regions was observed grossly and histologically. No severe adverse events associated with HIFU were reported for any of the cats, although mild-to-moderate thermal skin injuries occurred in two of three cases. The time to surgical resection following HIFU deviated from the original trial protocol for two cats. Surgical timelines were amended due to the progression of clinical signs attributable to the underlying disease and clinical schedule constraints. One patient exhibited pain associated with its rapidly growing mass, and the owner had limited availability for follow-up visits. Another patient experienced progressive anemia prior to HIFU treatment. A blood transfusion was administered to help decrease the risk of cardiovascular decompensation during anesthesia. The patient then developed a mild transfusion reaction manifested by an increase in body temperature and was transported to the intensive care unit (ICU) for overnight monitoring. As a result, surgical resection was performed one day after HIFU treatment for both patients. For both cats, clinical sign onset was prior to HIFU treatment and resolved following surgical removal and supportive care.

The treatment parameters used in this trial were adapted from our group’s completed study demonstrating the safety and feasibility of HIFU for canine STS [21]. In the canine study, gradable thermal injuries to the skin were observed in some patients, often in dogs with a hair type which was more challenging to remove. As a result, additional efforts were made to ensure complete hair removal for all cats in the current trial, but cutaneous thermal injury remains an important safety consideration for HIFU in cats. In the present trial, erythema of the skin overlying the treated mass was observed in all three cats, and two cats developed self-limiting cutaneous changes consistent with first-degree and superficial second-degree burns. These injuries did not require clinical intervention prior to planned surgical resection; however, the short interval between HIFU treatment and surgery may have limited assessment of the full evolution of thermal injury, as gross evidence of burns can become more apparent over subsequent days. Because feline STS may be superficial and located close to the skin or other critical structures, future studies should further evaluate strategies to reduce skin heating and should more fully characterize the incidence, severity, and clinical significance of HIFU-associated cutaneous injury.

In all three patients, HIFU treatment was successfully applied and monitored using real-time US imaging. After surgical resection, ablation zones were visible grossly and characterized by foci of tissue softening, suggestive of necrosis. Coagulative and lytic necrosis with varying degrees of hemorrhage and fibrin deposition were observed microscopically in treated areas. Notably, there was also a mixture of coagulative and lytic necrosis in pre-treatment and untreated tumor samples, likely attributable to standard tumor hypoxia. While solid portions of the tumor were targeted when possible in this study and areas of necrosis and hemorrhage were more extensive in treated sections, preexisting tumor necrosis may confound interpretation of the extent of HIFU-induced damage. For future studies, a CT scan one day post-treatment may allow for a better delineation of HIFU-treated areas from regions of existing tumor hypoxia, which can then be correlated to the grossly visible damage.

The secondary objective of this pilot study was to characterize the acute immunological responses following partial HIFU ablation in cats with STS. There was a statistically significant decrease in the level of MCP-1 detected after HIFU, but no discernable differences between pre- and post-treatment levels for the other analyzed cytokines. MCP-1, also known as CCL2, is involved in monocyte chemoattraction and immune cell trafficking [33]. Prior studies have demonstrated that MCP-1 accelerates tumor growth and metastasis via angiogenesis; therefore, a decrease in MCP-1 after HIFU treatment could represent decreased angiogenesis [34]. The exact roles of MCP-1 as well as many other cytokines and chemokines in cancer immunology, however, are complex and have yet to be fully elucidated. Outside of MCP-1, no statistically significant differences between pre- and post-treatment cytokine concentrations were observed. Additionally, no changes in the number or distribution of CD3-, CD79a-, or IBA1-positive cells were observed between samples in this study, suggesting that few lymphocytes or macrophages were recruited to the treatment site following HIFU.

The limited immunologic changes observed in this pilot study should be interpreted cautiously. The absence of broad serum cytokine changes or increased CD3-, CD79a-, or IBA1-positive immune cell infiltration does not necessarily indicate that feline STS are non-responsive to HIFU-induced immune modulation. Rather, several factors may have limited detection of an immune response in this cohort. First, the small sample size substantially limited statistical power and increased the influence of individual patient variability. Second, only partial tumor ablation was performed, and the treated volume may have been insufficient to generate a measurable local or systemic immune response. Third, two of three cats underwent tumor resection one day after HIFU, which may have been too early to detect late innate or adaptive immune cell recruitment. Finally, feline STS, including feline injection site sarcomas, may differ biologically from canine STS with respect to baseline inflammation, tumor microenvironment composition, and response to ablation. Therefore, the limited immune changes observed in this study cannot be attributed to a single mechanism. Future studies incorporating larger cohorts, greater treated tumor volumes, serial blood sampling, delayed tissue collection, and more comprehensive immune profiling will be needed to determine whether HIFU induces clinically meaningful immunomodulation in feline STS.

Importantly, prior studies indicate HIFU ablation has the potential to enhance host antitumor immunity [12,13,14,35,36,37]. One study in a murine model of sarcoma saw a significant increase in tumor-infiltrating lymphocytes after HIFU treatment as compared to untreated mice [7]. In our group’s previous canine STS HIFU study, there was a clear demarcation between treated tissue and non-treated tissue and a subjective increase in CD3+ immune cells along the zone of transition and within the ablation zone [21]. Additionally, there was a >2-fold increase in multiple genes associated with pro-inflammatory cytokine signaling in post-treatment samples, indicating an inflammatory response in the TME; no gene analysis was run for the cats due to the pilot nature and scope of the current study. However, in this canine clinical trial, surgical removal was performed 4–6 days post-treatment, a timepoint which may better capture the late innate and adaptive immune responses. Additionally, the canine trial included 15 dogs with STS, representing a notably larger population than the three cats with STS in the current study; future studies in felines should expand on this initial pilot study to yield more statistically robust evaluations of local and systemic immune responses after HIFU.

The average treated sites per tumor, mean delivered energy per tumor, and median requested power per pulse were higher in the canine study than the current feline HIFU treatments [21]. This suggests that larger treatment fields or higher thermal energies may be needed to observe similarly robust immune changes after treatment. One study investigating the development of metastatic disease following mechanical HIFU (histotripsy) treatment of rodent liver tumors suggests there may be a minimum ablation volume required to observe significant immune effects [38]. This trial observed a decrease in the amount of immune cell infiltration when histotripsy ablated less than 25% of the tumor volume compared to 50–75% of tumor volume. Histotripsy is a form of HIFU that achieves tumor ablation through mechanical destruction [39]. While this type of ablation may stimulate immune effects in a different way than thermal HIFU, these findings highlight the potential requirement for a minimum ablated tumor volume percentage to generate local immune changes, which may further explain the limited immunomodulation seen in this feline pilot study.

Our group is also exploring the use of histotripsy for the treatment of domestic animal tumors including feline STS, canine STS, canine osteosarcoma, and canine brain tumors and has demonstrated histotripsy’s immunogenic potential for each of these indications [40,41,42,43,44]. In a pilot study using histotripsy in three cats with STS, there was an increase in IBA1-positive cells in treated tumor tissues [40]. In this trial, tumors were resected 3–6 days after histotripsy, which could explain the observed increase in macrophages. However, there could also be an intrinsic difference in the type and amplitude of immunomodulation induced by thermal versus mechanical HIFU, which warrants further exploration. Non-thermal ablation modalities such as histotripsy are traditionally considered to be more immunogenic [45], but thermal ablation modalities such as HIFU have also shown promising immunomodulatory changes and warrant further exploration [12,13,14,35,36,37]. Future studies are needed to fully assess the relationship between the immunostimulatory potential of HIFU, the treatment volume, and the time elapsed between HIFU treatment and tumor removal in feline patients with STS.

Overall, the results of this study suggested that HIFU was able to achieve safe and effective tumor ablation in three cats diagnosed with spontaneously occurring soft tissue sarcoma, with the potential for immunomodulation. Complete surgical excision remains the standard-of-care treatment for feline STS, with radiation therapy commonly considered as an adjunctive local therapy when complete margins are not achievable or when recurrence risk is high. The present study was not designed to compare HIFU with standard-of-care treatment outcomes, and the limitations of a pilot trial preclude conclusions regarding local control, recurrence, disease-free interval, or survival. At present, these data do not support HIFU as a replacement for surgery or radiation therapy in cats with STS. In this pilot trial, HIFU was intentionally evaluated before standard-of-care surgical excision so that initial safety and feasibility could be assessed and treated tumor tissue could be examined histologically. These findings support the technical feasibility of applying HIFU to feline STS and demonstrate histologic evidence of tumor ablation within targeted regions. The potential clinical applicability of HIFU will require larger prospective studies designed to determine whether HIFU can improve margin control, reduce viable tumor burden prior to surgery, facilitate less aggressive surgery, or provide local treatment for tumors that are incompletely resectable or not amenable to conventional local therapies.

This study had several important limitations, including the absence of a control group and the small sample size. The objective of this pilot study was to evaluate the initial safety and feasibility of HIFU treatment in cats with STS prior to surgical excision. Because HIFU had not previously been reported in cats, a small prospective pilot study was considered to be an appropriate first step before larger controlled studies were pursued. Another limitation of this pilot study was that enrollment was restricted to cats with tumors considered to be surgically resectable. This criterion was selected because HIFU had not previously been evaluated in cats, and surgical resection after treatment allowed patients to receive standard-of-care local therapy while also permitting histologic evaluation of the treated tumor region. As a result, these findings are most applicable to cats with resectable peripheral STS and should not be extrapolated to cats with more advanced, unresectable, or non-surgical disease without further investigation. While this study was limited by its small patient population, it provided important clinical evidence of the safety and feasibility of HIFU for feline STS and supports future studies to fully characterize HIFU’s utility as a non-invasive ablative and potentially immunostimulatory therapy in cats. Enrollment in single-institution feline STS trials can be challenging, particularly when eligibility is restricted to cats with resectable, externally accessible tumors appropriate for device-based intervention; future adequately powered studies may therefore require extended enrollment periods or multi-institutional collaboration. A final challenge of this study was the logistical consideration of patient body size for ablative therapies. Because cats are generally smaller than dogs, the ablation was limited to a smaller treatment site, and alterations in treatment protocols and devices may be needed. This introduced additional complexity when comparing the findings from this pilot study to those from the previous canine STS trial, particularly when evaluating the immune response, and highlights the potential need for tailored HIFU devices for different species and applications in veterinary medicine.

## 5. Conclusions

The results of this pilot study provide preliminary evidence of HIFU’s potential as a non-invasive and possibly immunomodulatory treatment for soft tissue sarcoma (STS) in cats. HIFU was well tolerated in three feline patients with STS, and gross and histological analyses reveal effective ablation in the targeted tumor regions. Additionally, there was a measurable change in the level of serum MCP-1 after HIFU treatment, although the clinical significance of this finding is unclear due to the study’s small sample size (*n* = 3) and short follow-up. Future studies investigating HIFU for the complete ablation of feline STS as well as the optimization of the immunological response after treatment are warranted.

## Figures and Tables

**Table 1:** Patient demographics, tumor characteristics, and outcomes.

Patient	Breed	Age (yrs)	Gender	Tumor Details	Tumor Location	Tumor Size (LD) (cm)	Follow-Up
1	DSH	3	FS	PD: Grade II STS ST: Fibrosarcoma	Left lateral hind limb	5.3	Monitoring
2	DSH	10	FS	PD: Grade III STS ST: Fibrosarcoma	Right lateral hind limb	11.8	Single-agent doxorubicin
3	Siamese	15	MN	PD: Grade II STS ST: Fibrosarcoma	Left lateral thorax	4.4	Monitoring

Patient information ordered by the date of HIFU treatment, including tumor characteristics and outcomes. Abbreviations: DSH = domestic shorthair, MN = male neutered, FS = female spayed, PD = primary diagnosis, ST = subtype, LD = longest diameter.

**Table 2:** Serum cytokine concentrations pre-treatment (tx) and 1 day post-treatment.

Analytes	Patient 1		Patient 2		Patient 3		Mean	
	Pre-Tx (pg/mL)	Post-Tx (pg/mL)	Pre-Tx (pg/mL)	Post-Tx (pg/mL)	Pre-Tx (pg/mL)	Post-Tx (pg/mL)	Pre-Tx (pg/mL)	Post-Tx (pg/mL)
*Fas*	31.83	27.32	0	0	47.42	49.01	26.42	38.17
*Flt-3L*	85.62	82.7	148.03	117.55	161.17	158.89	131.61	119.71
*GM-CSF*	28.6	21.39	0	0	22.81	22.1	17.14	14.50
*IFN-γ*	811.61	746.06	148.75	143.95	784.75	740.12	581.70	543.38
*IL-1β*	160.38	148.15	0	0	299.7	300.67	153.36	149.61
*IL-2*	104.58	89.8	19.96	0	31.12	27.37	51.89	39.06
*IL-12*	141.83	154.82	574.34	633.5	245.49	374.01	320.55	387.44
*IL-13*	100.64	97.48	15.24	15.24	83.1	90.51	66.33	67.74
*IL-4*	2130	1917	304.83	251.03	1917	1882	1450.61	1350.01
*IL-6*	1074	805.94	140.36	129.28	646.97	612.62	620.44	515.95
*IL-8*	91.53	84.79	118.21	113.75	73.1	73.1	94.28	90.55
*KC*	17.25	18.77	0	2.86	4.06	3.93	7.10	8.52
*SDF-1*	3337	2652	2315	1178	4906	4965	3519.33	2931.67
*RANTES*	78.92	72.53	78.34	79.5	91.03	89.89	82.76	80.64
*SCF*	358.71	330.38	201.81	184.53	382.07	401.7	314.20	305.54
*MCP-1*	7882	7599	3422	3205	6795	6404	**6033**	**5736**
*TNFα*	247.1	194.55	0	0	203.2	177.4	150.10	123.98
*IL-18*	958.92	865.62	0	0	209.46	195.25	389.46	353.62

A significant decrease in MCP-1 (bold; two-tailed *p* = 0.0279) was measured after HIFU treatment. No other statistically significant differences were observed. The analytes below the minimum detection limit of the assay were excluded from the table.

## Data Availability

To protect client and patient privacy, the raw data supporting the conclusions of this pilot study are available only upon request from the corresponding author.

## Abbreviations

The following abbreviations are used in this manuscript:

AE	Adverse event
ACVP	American College of Veterinary Pathologists
ACVS	American College of Veterinary Surgeons
CBC	Complete blood count
CT	Computed tomography
fISS	Feline injection site sarcoma
H&E	Hematoxylin and eosin
HIFU	High-intensity focused ultrasound
MCP-1	Monocyte chemoattractant protein-1
MRI	Magnetic resonance imaging
STS	Soft tissue sarcoma
TME	Tumor microenvironment
US	Ultrasound
VCOG-CTCAE	Veterinary Cooperative Oncology Group—Common Terminology Criteria for Adverse Events
VTU	Visualization and treatment unit
